# Discovering Diverse Roles of Peroxidases and Catalases in Photosynthetic and Non-Photosynthetic Eukaryotes

**DOI:** 10.3390/antiox11122337

**Published:** 2022-11-25

**Authors:** Marcel Zámocký

**Affiliations:** 1 Laboratory for Phylogenomic Ecology, Institute of Molecular Biology, Slovak Academy of Sciences, Dúbravská Cesta 21, SK-84551 Bratislava, Slovakia; marcel.zamocky@savba.sk; Tel.: +421-2-5930-7481; 2 Department of Chemistry, Institute of Biochemistry, University of Natural Resources and Life Sciences, Muthgasse 18, A-1190 Vienna, Austria

This Special Issue of *Antioxidants*, dedicated to “The Role of Peroxidases and Catalases in Photosynthetic and Non-photosynthetic Eukaryotes“, was accomplished with the contribution of five original research articles and two detailed reviews. Although this broad research topic has been the subject of investigation for a significant length of time, from various aspects, some unifying approaches and important general conclusions from the interpretation of diverse results are urgently needed in the scientific literature. This Special Issue therefore delivers results a practical overview on antioxidant enzymes, mainly from following three kingdoms of the domain Eukarya: Fungi, Plantae and Excavata.

Catalases have already been the focus of intensive studies for more than a century. However, new and important facts have been revealed in recent findings. Hansberg et al. [1] discovered a new functional domain present in large subunit heme catalases possessing independent molecular chaperone activity. This C-terminal domain has significant similarity with the DJ-1/PfpI superfamily.

Both enzymatic activities here investigated, namely, peroxidase with ascorbate as electron donor and catalase, were subjected to detailed analyses in *Lemna minor* plants within the acquired tolerance of this aquatic freshwater plant to antibiotics amoxicillin, ciprofloxacin, and erythromycin, which pose a global contamination problem in various environments [2]. The resulting effects, tested with specific inhibitors, were different for peroxidase and catalase activities. APx is significant in avoiding oxidative damage caused by amoxicillin, whereas catalase decreased the deleterious effects of ciprofloxacin. Both of these enzyme activities had a synergic effect in supporting the tolerance of plant to erythromycin.

Chmelová et al. [3] have demonstrated that genes for unique catalases from parasitic Trypanosomatidae have been transferred in their genomes by means of horizontal gene transfer from Proteobacteria. Among these genes, the authors discovered a gene coding for a unique cyanide-resistant heme catalase in the genus *Blastocrithidia*, caused by a single mutation in the main substrate channel.

Lignin peroxidases from the largest peroxidase–catalase superfamily [4] are already well known for their involvement in the highly efficient degradation of wood lignin. Sanchez-Ruiz et al. [5] have discovered a new variant from a soil-inhabiting mushroom *Agrocybe pediades*. Similarly to many other lignin peroxidases, it has a highly conserved heme pocket and a solvent-exposed tryptophan that represents the oxidation site for lignin and other high-redox-potential substrates.

Another important member of the peroxidase–catalase superfamily is ascorbate peroxidase. Besides classical enzymes efficiently oxidizing the ascorbate, unusual subsets of their homologs were identified by Lazzarotto et al. [6]. Whereas the ascorbate peroxidase-related subfamily harbors all catalytic residues for general peroxidatic activity, they do not contain specific amino acid residues known to bind the ascorbate. In contrast, the ascorbate peroxidase-like subfamily even also lacks amino acid residues that are essential for peroxidation. The performed phylogenetic analysis revealed the origin of these peculiar proteins and detected a mutational drift towards neofunctionalization of their catalytic sites.

In his review [7], Hansberg focuses on the substantial features of heme catalases. He introduces a specific gate valve system present in the final section of the main substrate channel. Additionally, the conversion of heme *b* to heme *d* in large subunit catalases is discussed, and the involvement of singlet oxygen in this and other post-translational modifications within catalases is addressed. Finally, the presence of an additional C-terminal domain selectively in large subunit catalases with proven Hsp-31-type chaperone activity is documented here.

Plant glutathione peroxidases are in contrast to their animal counterparts’ non-heme and non-seleno monomeric proteins, as reviewed by Bela et al. [8]. They generally utilize thioredoxin as a reducing agent for the conversion of hydrogen peroxide or organic peroxides. The authors also introduce here the group of GPX-like enzymes that not only protect plant cells from oxidative damage, but are also crucial components of plant development and growth.

All data and results presented in this Special Issue on various types of enzymes reacting with peroxides reveal surprising diversity and flexibility that have evolved in various gene families for the efficient removal of reactive oxygen species and involvement in essential physiological processes.
